# Periodontitis and peripheral artery disease: a mini-review

**DOI:** 10.3389/froh.2026.1728706

**Published:** 2026-01-23

**Authors:** Domenico De Falco, Sergio Zacà, Margot Ringold, Francesca Sodero, Domenico Angiletta, Massimo Petruzzi

**Affiliations:** 1 Interdisciplinary Department of Medicine, University of Bari, Bari, Italy; 2 Vascular and Endovascular Surgery-Department of Emergency and Organs Transplantation, “Aldo Moro” University of Bari School of Medicine, Bari, Italy

**Keywords:** acute limb ischemia, cardiovasculardisease, periodontal disease, periodontitis, peripheral artery disease

## Abstract

Periodontitis is a inflammatory disease characterized by progressive loss of periodontal attachment and alveolar bone, leading to tooth mobility and eventual tooth loss. Periodontal disease affects about half of U.S. adults. Epidemiologic evidence links periodontitis to increased incidence of cardiovascular disease (CVD). Peripheral artery disease (PAD) is common in adults aged ≥65 years and is associated with substantially increased cardiovascular risk. It is an atherosclerotic condition that shares major risk factors, diabetes, smoking, older age, hypertension, and chronic kidney disease. Although the link between periodontitis and CVD is well established, comparatively few studies have examined PAD specifically. This mini-review synthesizes recent studies on periodontitis and PAD. Across heterogeneous designs and populations, most reports support a modest association, which appears stronger in the presence of shared risk factors (e.g., diabetes, smoking) and with more severe periodontal involvement. Several studies have detected oral pathogens or pathogen-specific antibodies (e.g., *Porphyromonas gingivalis*, *Treponema denticola*) in patients with PAD and report higher circulating inflammatory mediators. Nonetheless, substantial heterogeneity in populations, exposure/outcome definitions, and confounding control, and the likelihood of residual confounding, limit causal inference. Large, well-designed prospective studies with standardized periodontal phenotyping and rigorous adjustment (including attention to temporal trends in dental extraction practices) are needed to define effect magnitude and clinical relevance.

## 1 Introduction

Periodontal diseases (PD) encompasses two related but distinct conditions: gingivitis and periodontitis. Gingivitis is an inflammation of the gingival tissues induced by dental plaque accumulation and represents a localized, typically reversible immune response (1). Its course can be modified by factors such as smoking, medications, and hormonal fluctuations (puberty and pregnancy) (2, 3). Gingivitis may progress to periodontitis, a more severe disease where the host's immune–inflammatory response contributes to loss of periodontal ligament, alveolar bone, and other supporting tissues. Periodontitis is clinically more significant, as it can result in tooth mobility and tooth loss (2, 4, 5). PD affects approximately 50% of the U.S. (United States) population, although prevalence estimates vary across studies (6–8). It predominantly involves adults over 30 years of age, with clinical presentations ranging from moderate to severe (2, 9, 10). The association between PD and atherosclerotic cardiovascular disease (CVD) has long been recognized (11, 12).

Transient bacteremia is a normal event in which routine dental activities, chewing, toothbrushing, periodontal probing, or extractions, allow oral bacteria to enter the bloodstream (13). In healthy individuals, these bacteria are cleared within minutes by the innate immune system (14). Leukocytes are relatively ineffective at capturing pathogens in rapidly flowing blood; instead, the complement system opsonizes bacteria with C3b, which binds CR1 on erythrocytes and immune cells (14). Erythrocytes, by virtue of their abundance, sequester and damage bacteria and deliver them to the liver and spleen, where Kupffer cells and splenic macrophages phagocytose them (14). When key complement components (C3/C5) are absent, this clearance fails and bacteremia can become overwhelming (14).

Specific periodontal bacterial strains can evade normal bloodstream clearance in some patients through complement-evasion mechanisms and may contribute to atherogenesis (15). In particular, *Aggregatibacter actinomycetemcomitans*, *Fusobacterium nucleatum*, *Porphyromonas gingivalis*, *Tannerella forsythia*, and *Treponema denticola* can disseminate hematogenously and invade distant sites (14). *P. gingivalis* is the organism most frequently detected in bacteremias of oral origin and has been identified in amniotic fluid, placenta, brain, cerebrospinal fluid, and vascular tissues (14, 15).

The presence of oral bacteria within atheromatous plaques has been extensively documented in patients with acute myocardial infarction and stroke (16–19). In addition, the systemic increases in inflammatory mediators, such as Interleukin-6 (IL-6), Interleukin-1β (IL-1β), and Tumor Necrosis Factor-α (TNF-α), in patients with CVD suggests that chronic infection may play an important role (20). As a chronic inflammatory condition, PD can elicit local and systemic host immune responses, provoke transient bacteremias, and drive the release of inflammatory mediators (including interleukins and TNF-α) that injure the vascular endothelium and, over time, contribute to the development of CVD (9, 20, 21).

Peripheral Artery Disease (PAD) is a condition commonly linked to atherosclerosis that affects up to 20% of U.S. adults aged ≥65 years (22, 23). This occurs when atherosclerotic plaques accumulate within peripheral arteries (24). PAD causes arterial obstruction, which can lead to ischemic ulcers, gangrene, and, in some cases, amputation (25–27). PAD is associated with markedly increased cardiovascular mortality, conferring a three- to sixfold higher risk (28). Several risk factors for PAD include diabetes, smoking, advanced age, hypertension, and chronic kidney disease (29). Although the association between PD and CVD is well established, much less attention has been given in the literature to the relationship between PD and PAD (9, 30–35). The objective of this mini-review was to examine and summarize the literature on the association between PD and PAD, with particular emphasis on the most recent studies.

## 2 Materials and methods

This mini literature review was conducted using the MEDLINE/PubMed and Scopus databases, applying the following search strategy: (“Periodontal Diseases” OR periodontitis OR “periodontal disease” OR “periodontal condition”) AND (“Peripheral Arterial Disease” OR “Peripheral Vascular Diseases” OR “peripheral artery disease” OR “peripheral arterial disease” OR PAD OR “peripheral vascular disease”). Two authors (D.D.F. and S.Z.) independently screened titles and abstracts for eligibility; discrepancies were resolved by discussion and, when necessary, by consulting a third reviewer (M.P.). All articles published between 2000 and December 2025 were considered, and only studies published in English were included. Eligible evidence included primary studies in humans (observational studies and interventional/clinical trials), animal studies, and *in vitro* studies. Secondary sources (systematic reviews, narrative reviews, and book chapters) were also considered to contextualize the available evidence.

## 3 Pathogenetic considerations

Systemic inflammation can be regarded as the common thread linking periodontal inflammation to peripheral atherothrombosis. Beyond direct vascular effects, driven by episodic bacteremia and endothelial activation, PD may also contribute to PAD indirectly by worsening the systemic metabolic milieu. In particular, it may promote insulin resistance and poorer glycemic control, foster a more atherogenic lipid profile, and increase prothrombotic tendency. Through these pathways, endothelial injury and plaque vulnerability may be amplified, especially in individuals who already carry high-risk conditions such as diabetes and/or chronic kidney disease (36, 37). From a translational Predictive, Preventive, and Personalized Medicine (3PM) perspective, periodontal status could therefore be viewed as a modifiable marker of systemic inflammatory burden and overall metabolic risk (36). A cross-organ framework of this kind has been proposed in other vascular–immune contexts (for example, the oral–ocular interface) and supports the rationale for integrating oral health into personalized vascular risk stratification and prevention strategies (36, 38).

PAD can be interpreted as the peripheral manifestation of a low-grade but prolonged atherothrombotic process in which systemic inflammation, endothelial dysfunction, and hemostatic–lipid imbalances interact along a continuum (39). Within this framework, PD is associated with a sustained increase in circulating inflammatory mediators (e.g., IL-6, TNF-α, C-Reactive Protein—CRP, Serum Amyloid A and microbial components -lipopolysaccharide, which may contribute to endothelial activation and dysfunction (40–42).

In line with this hypothesis, a 2008 case-control study by Chen et al. (*n* = 25 PAD) suggested that patients with PD may have an approximately fivefold increased risk of PAD compared with individuals without PD (43). Importantly, the detection of periodontal bacterial strains in PAD patients was accompanied by signals consistent with heightened systemic inflammation, including elevated IL-6 and TNF-α (43). Moreover, higher IgG levels against Porphyromonas gingivalis and Treponema denticola were reported in PAD patients, and these bacteria were identified in atherosclerotic specimens, supporting a potential microbial/immune contribution to atherogenesis (43).

Mechanistically, such a cytokine-rich milieu provides a plausible upstream driver for the endothelial phenotype described below, consistent with up-regulation of Intercellular Adhesion Molecule-1 (ICAM-1) and Vascular Cell Adhesion Molecule-1 (VCAM-1) and selectins and, consequently, increased monocyte adhesion and diapedesis within lower-limb arteries (44, 45). Once in the intima, monocytes differentiate into macrophages and, in the presence of modified/oxidized Low Density Lipoprotein (LDL) and Toll-Like Receptors (TLR) dependent cues, can promote lipid uptake and become foam cells (39, 44). This fuels plaque growth and shifts the microenvironment toward the Matrix Metalloproteinase (MMP) release, which thin the fibrous cap and reduce plaque stability (39). In addition, there may be a potential contribution of recurrent bacteremias from periodontal pathogens (vascular *“*priming”) and autoimmune mechanisms that amplify local immune activation (45–47). These proposed mechanism remain speculative and should be validated in longitudinal and interventional studies.

In parallel, an atherogenic lipid profile and a prothrombotic state favor intraplaque thrombosis and microembolization (39). Functionally, oxidative stress and Nuclear Factor-*κ*B activation reduce nitric oxide bioavailability, limiting vasodilation and collateral arteriogenesis; the result is a peripheral vascular bed more vulnerable to chronic ischemia (39). In advanced stages, hypoxia and persistent inflammation impair tissue repair, predisposing to ischemic ulcers, gangrene, and, in refractory cases, amputation (39). The impact of each step is modulated by shared risk factors (smoking, diabetes, dyslipidemia, hypertension) and by genetic susceptibility, which together account for the clinical variability observed among patients (39).

## 4 Epidemiologic and biobank evidence linking periodontitis to peripheral artery disease

### 4.1 Prospective cohort studies

The association between PD and PAD had long been discussed in the literature as reported in Table 1. As early as 1998, Mendez et al., in a prospective study, first reported an association between chronic PD and PAD (48). However, the authors were unable to clarify the mechanism underlying this relationship (48). In the 2017 classification, the former entities “chronic periodontitis” and “aggressive periodontitis” were consolidated into a single diagnostic category termed “periodontitis” (49).

**Table 1:** Summary of epidemiological/clinical human studies on periodontitis and peripheral arterial disease (PAD) discussed in this review.

Study	Design & population	Definitions (PD; PAD)	Main association	Adjusted confounders (fully adjusted)
Prospective cohort studies				
Mendez et al. (48)	Design: Prospective cohort Cohort/Study: Normative Aging Study; Dental Longitudinal Study Country: US Period: 1961–1985 Follow-up: 25–30 years Population: US veterans; initially healthy; 80 incident PVD cases vs. 1,030 controls Age/Sex: NR/M	PD: Clinically significant PD at baseline (clinical exam; per study definition) PAD: Incident PVD during follow-up	Adjusted risk increment 2.27 (95% CI: 1.32–3.90) for baseline PD	Age; BMI; family history of heart disease; smoking exposure
Hung et al. (50)	Design: Prospective cohort Cohort/Study: Health Professionals Follow-up Study Country: US Period: 1986–1998 Follow-up: 12 years Population: 45,136 male health professionals free of CVD at baseline; 342 incident PAD cases Age/Sex: 45–70 years at baseline; M	PD: History of PD (self-report) and incident tooth loss during follow-up PAD: Incident PAD (cohort case ascertainment)	RR: 1.41 (95% CI: 1.12–1.77) for PD history; RR: 1.39 (1.07–1.82) for any tooth loss; RR: 1.88 (1.27–2.77) for tooth loss among men with PD	Age; smoking (status/amount); alcohol intake; BMI; physical activity; diabetes; hypertension; aspirin use; multivitamin use; dentist status
Muñoz-Torres et al. (51)	Design: Prospective cohort Cohort/Study: Nurses’ Health Study Country: US Period: 1992–2008 Follow-up: 16 years Population: 79.663 women; 277 incident PAD cases during follow-up Age/Sex: mean age 58 years; F	PD: Incident tooth loss and baseline number of teeth PAD: Incident PAD during follow-up	Incident tooth loss: HR: 1.31 (95% CI: 1.00–1.71); baseline number of teeth: no dose-response relationship	Age; smoking; diabetes; hypertension; high cholesterol; aspirin use; family history of MI; BMI; alcohol; physical activity; postmenopausal hormone use; vitamin E/D; multivitamin; calcium
Arsiwala et al. (9)	Design: Prospective cohort Cohort/Study: ARIC Country: US Period: 1996–1998 Follow-up: Median 20.1 years Population: Community-based cohort; 360 incident PAD cases (3.6%) Age/Sex: mean age 62.8 ± 5.6 years; 44.3% M	PD: Self-reported tooth loss because of PD; history of periodontal treatment; PD diagnosis; plus clinical PD (CDC/AAP) in subset PAD: Incident PAD defined by hospital admission diagnosis or procedures	HR: 1.54 (95% CI: 1.20–1.98) for tooth loss due to PD; HR: 1.37 (1.05–1.80) for periodontal treatment history; HR: 1.38 (1.09–1.74) for periodontal diagnosis; clinical severe PD not significant in fully adjusted model [e.g., HR: 1.53 (0.94–2.50)]	Age; sex; race; education level; diabetes; smoking (pack-years); systolic blood pressure; anti-hypertensive medication; total cholesterol; HDL; lipid-lowering therapy; history of cardiovascular disease
Cross-sectional studies				
Ahn et al. (52)	Design: Cross-sectional Cohort/Study: Yangpyeong county cohort Country: Korea Period: 2010–2014 Follow-up: N/A (cross-sectional) Population: 1,343 dentate adults aged >40 years Age/Sex: Age >40; M/F	PD: RABL on panoramic radiograph; severe: ≥2 non-adjacent interproximal sites with RABL ≥6 mm (moderate: ≥4 mm) PAD: ABI ≤1.0 by Doppler	Severe PD vs. normal periodontal status: aOR: 2.03 (95% CI: 1.05–3.93)	Age; sex; education; tooth loss; smoking; drinking; exercise; obesity; triglycerides; HDL; LDL; hs-CRP; diabetes; hypertension
Aoyama et al. (53)	Design: Cross-sectional (hospital-based) Cohort/Study: Tokyo Medical and Dental University Hospital Country: Japan Period: NR Follow-up: N/A (cross-sectional) Population: PAD *n* = 34 vs. non-PAD *n* = 956 Age/Sex: Adults; M/F	PD: Clinical periodontal measures (teeth number, PPD, BOP, CAL) and edentulism PAD: Presence vs. absence of PAD (clinical diagnosis); ABI measured in PAD context	Descriptive comparisons: PAD patients had more missing teeth and higher edentulism; higher serum inflammatory factors vs. non-PAD	Not adjusted (smoking; diabetes; hypertension; dyslipidemia)
Çalapkorur et al. (29)	Design: Cross-sectional Cohort/Study: Single-center Country: Turkey Period: NR Follow-up: N/A (cross-sectional) Population: 60 patients classified by ankle-brachial index (PAD vs. non-PAD) Age/Sex: Adults; M/F	PD: Clinical periodontal examination (parameters compared between groups) PAD: ABI-based grouping (test PAD vs. control)	No significant differences in clinical periodontal parameters between PAD and non-PAD groups	Age; sex; diabetes; hypertension; BMI
Jacobi et al. (34)	Design: Population-based cohort (cross-sectional analysis) Cohort/Study: Hamburg City Health Study Country: Germany Period: 2016 onward (baseline recruitment) Follow-up: N/A (cross-sectional analysis) Population: Subset *N* = 3,271 with complete vascular and oral exam Age/Sex: 45–74 years; M/F	PD: Full-mouth exam at 6 sites/tooth (PPD, gingival recession, CAL, BOP); graded moderate/severe PAD: PAOD if ABI ≤0.9, ABI >1.4, or history of peripheral artery revascularization and/or amputation	Severe PD independently associated with PAOD: OR: 1.265 (95% CI: 1.006–1.591; *p* = 0.045)	Age; sex; diabetes; education; smoking; hypertension; hs-CRP
Yu et al. (38)	Design: Biobank cross-sectional Cohort/Study: U.S. Veterans Affairs Million Veteran Program Country: US Period: Jan 2011–Sep 2021 Follow-up: N/A (cross-sectional) Population: 154,167 US veterans Age/Sex: mean age 65.5 years; 92% M	PD: ICD-coded PD and tooth loss; (self-reported oral health) PAD: PVD as cardiometabolic comorbidity using ICD codes	Tooth loss associated with PVD: OR: 1.82 (*p* < 0.001)	Demographics; socioeconomic status; cardiovascular covariates; inflammatory covariates (neutrophil/lymphocyte counts; CRP; albumin)
Administrative/claims-based studies				
Cho et al. (33)	Design: Nationwide matched cohort Cohort/Study: NHIS-HEALS Country: Korea Period: Jan 2003–Dec 2014 Follow-up: Up to 12 years Population: National Health Insurance screening cohort; periodontitis vs. matched controls Age/Sex: 40–79 years; M/F	PD: ICD-10 K05.2-K05.3; required ≥2 principal diagnostic codes PAD: ICD-10 I70, I70.0, I70.2-I70.3, I70.8-I70.9, I74.2-I74.5; required ≥2 principal diagnostic codes	HR: 1.15 (95% CI: 1.07–1.23); incidence per 1,000 person-years: 2.40 (PD) vs. 2.08 (controls)	Age; sex; BMI; smoking; alcohol; exercise; hypertension; diabetes; dyslipidemia; ischemic heart disease; stroke; medication use (DM/HTN meds; statins; antithrombotics)
Aarabi et al. (30)	Design: Retrospective health insurance claims analysis Cohort/Study: BARMER Country: Germany Period: Jan 2012–Dec 2016 Follow-up: N/A (retrospective) Population: 70,944 hospitalized symptomatic PAD patients; outcomes: intermittent claudication vs. CLTI Age/Sex: Adults; M/F	PD: Prior periodontal treatment (non-surgical/surgical) identified via ambulatory coding PAD: ICD-10 codes for intermittent claudication and CLTI	Not being treated for PD associated with CLTI (vs. intermittent claudication): OR: 1.97 (95% CI: 1.83–2.13)	Age; sex; diabetes
Yeh et al. (37)	Design: Nationwide retrospective cohort Cohort/Study: LHID2000 Country: Taiwan Period: 2000–2018 Follow-up: N/A (retrospective) Population: After propensity-score matching: 357,106 periodontitis vs. 357,106 non-periodontitis Age/Sex: Adults; M/F	PD: Newly diagnosed PD identified via claims PAD: PAOD identified via claims	Adjusted HR: 1.03 (95% CI: 1.01–1.06) for PD vs. non-PD; ≥1 dental scaling procedure reduced risk	Age; sex; coronary artery disease; diabetes; hypertension; hyperlipidemia; chronic kidney disease; COPD; asthma; ischemic stroke; hemorrhagic stroke; dental scaling

AAP, American Academy of Periodontology; ABI, ankle-brachial index; aHR, adjusted hazard ratio; aOR, adjusted odds ratio; ARIC, Atherosclerosis Risk in Communities (study); BMI, body mass index; BOP, bleeding on probing; CAD, coronary artery disease; CAL, clinical attachment level; CDC, Centers for Disease Control and Prevention; CI, confidence interval; CKD, chronic kidney disease; CLTI, chronic limb-threatening ischemia; COPD, chronic obstructive pulmonary disease; CRP, C-reactive protein; CVD, cardiovascular disease; DBP, diastolic blood pressure; DM, diabetes mellitus; HDL, high-density lipoprotein; HDL-C, high-density lipoprotein cholesterol; HR, hazard ratio; hs-CRP, high-sensitivity C-reactive protein; HTN, hypertension; ICD, International Classification of Diseases; ICD-10, International Classification of Diseases, Tenth Revision; ICD-9, International Classification of Diseases, Ninth Revision; IL-6, interleukin-6; LDL, low-density lipoprotein; LDL-C, low-density lipoprotein cholesterol; LHID, Longitudinal Health Insurance Database; MI, myocardial infarction; MMPs, matrix metalloproteinases; NHIS, National Health Insurance Service; NHIS-HEALS, National Health Insurance Service-Health Screening Cohort; NLR, neutrophil-to-lymphocyte ratio; OR, odds ratio; PD, periodontal disease; PAD, peripheral artery disease; PAOD, peripheral arterial occlusive disease; PPD, probing pocket depth; PSM, propensity score matching; PVD, peripheral vascular disease; RABL, radiographic alveolar bone loss; RR, relative risk; SBP, systolic blood pressure; SES, socioeconomic status; TC, total cholesterol; TG, triglycerides; TNF-α, tumor necrosis factor alpha; US, United States.

A 2003 prospective cohort study by Hung et al. followed 45,094 men aged 40–75 years for 12 years to assess the incidence of PAD (50). The study found that men who lost one or more teeth during the study period had a higher risk of developing PAD (multivariable-adjusted relative risk-RR: 1.39, 95% CI: 1.07–1.829). A history of PD also increased the risk of PAD (RR: 1.41; 95% CI: 1.12–1.77) (50). Loss of at least one tooth was associated with a 39% higher risk of PAD, and tooth loss among men who also had PD was linked to an almost twofold increase in PAD risk (RR: 1.88; 95% CI: 1.27–2.77) (50).

In a 2017 prospective analysis from the Nurses’ Health Study, Muñoz-Torres et al. examined whether tooth loss was associated with incident PAD (51). After excluding women with pre-existing CVD, 79,663 participants were followed for 16 years, during which 277 incident PAD cases were confirmed (51). In multivariable-adjusted Cox models, incident tooth loss (self-reported and updated over follow-up) was associated with a modest increase in PAD risk (hazard ratio-HR: 1.31, 95% CI: 1.00–1.71), whereas the baseline number of teeth did not show a clear dose–response relationship (51). Overall, the findings suggested a small but measurable association between deterioration in oral status and PAD, with potential influence from exposure misclassification and residual confounding (51).

In a recent large prospective cohort study conducted in the U.S. (2021), Arsiwala et al. followed nearly 10,000 participants for about 20 years (9). They observed a modest association between PD and subsequent risk of PAD (9). However, the association became significant in the presence of shared risk factors such as diabetes and smoking, and the authors suggested that oral inflammation may reinforce this relationship (9). The association was stronger among patients with severe PD, indicating that only severe PD might materially have influenced PAD risk (9). The study's strengths included its large sample size and long follow-up period (9).

### 4.2 Cross sectional studies

In 2016, Ahn et al. showed, in a Korean population sample, that PAD and severe PD were independently associated after adjustment for confounders including age, sex, education level, smoking, alcohol use, physical activity, central obesity, triglycerides, high-density lipoprotein (HDL), low-density lipoprotein (LDL), CRP, diabetes, and hypertension (52). The authors also confirmed the presence of bacterial groups in atherosclerotic plaques, including *Tannerella forsythia*, *Porphyromonas gingivalis*, and *Prevotella* (43).

Another study in a Japanese population found higher rates of edentulism and a greater number of missing teeth among individuals with PAD than among those without PAD (53). The study considered that, in Japan, PD was the leading cause of tooth loss (53). Furthermore, the authors contended that the degree of edentulism was the most reliable proxy for severe, irreversible PD (53). Unlike other periodontal indices, probing pocket depth (PPD), clinical attachment level (CAL), and bleeding on probing (BOP), edentulism was intrinsically stable and was not susceptible to short-term fluctuation (53). However, indications for tooth extraction had changed over time with advances in evidence-based dentistry and technology (53). This temporal trend should have been incorporated as a covariate in the analysis (53).

A dedicated systematic review conducted by Yang et al. (2018) emphasized that missing teeth could reflect an irreversible condition in the end stages of PD (21). In their meta-analysis, they confirmed the previously noted finding that patients with PAD typically had a greater number of missing teeth than participants without PAD (21). This result strengthened the hypothesis of a close relationship between PD and PAD (21).

Evidence on systemic inflammatory biomarkers linking PD to PAD remained controversial. In one study, Çalapkorur et al. found no differences in either systemic (e.g., CRP) or local (gingival crevicular fluid Pentraxin 3) inflammatory markers between PAD and non-PAD participants (29). The authors attributed this “flatness” to population heterogeneity (advanced age, multiple concomitant therapies, smoking/alcohol), which may have masked true differences. These findings are at odds with reports from other studies, such as the previously cited work by Chen et al. (43). In any case, important limitations persisted in the literature, including poor homogeneity of study populations and the inability to adjust for all systemic factors. Along the same lines, the meta-analysis by Yang et al. found no differences in CAL between patients with PAD and participants without PAD (21). The authors likewise concluded that this result might have been driven by heterogeneity among study groups and the limited number of relevant studies (21).

In a 2021 population-based analysis nested within the prospective Hamburg City Health Study, Jacobi et al. investigated whether PD was independently associated with PAD (34). The cross-sectional dataset included 3,271 adults aged 45–74 years who underwent both vascular and comprehensive periodontal examinations. PAD was ascertained using an ankle–brachial index <0.9 complemented by lower-limb vascular ultrasound and questionnaire-based clinical history, while periodontal status was derived from a full-mouth six-sites-per-tooth protocol (PPD, gingival recession, CAL, and BOP), with severity classified according to established criteria. In multivariable logistic regression models adjusting for key confounders (including age, sex, smoking, education, diabetes, hypertension), severe PD remained significantly associated with PAD (odds ratio-OR: 1.265; 97.5% CI 1.006–1.591; *p* = 0.045). The authors noted that participants with both conditions showed a more adverse cardiometabolic profile (e.g., higher prevalence of smoking and diabetes), underscoring the importance of rigorous adjustment, while the cross-sectional nature of the analysis limited causal and temporal inference.

Yu Y.H. et al. published in 2025 a cross-sectional observational study, conducted between 2011 and 2021, that included 154,167 U.S. veterans and investigated the relationship between oral health, inflammation, and cardiometabolic diseases (38). The results showed that, as self-rated oral health worsened, the prevalence of coronary artery disease, hypertension, heart failure, myocardial infarction, stroke, PAD, and diabetes increased steadily (38). In particular, compared with participants reporting excellent oral health, the multivariable-adjusted OR for PAD were 1.26 (95% CI: 1.14–1.39) for “good”, 1.56 (95% CI: 1.41–1.73) for “fair”, and 1.89 (95% CI: 1.70–2.10) for “poor” oral health (38). Similarly, mean neutrophil counts, and to a lesser extent lymphocyte counts, also increased (38). Clinical diagnoses of PD and tooth loss likewise showed a stronger association with PAD, with adjusted OR of 1.60 (95% CI: 1.51–1.69) for any PD and 1.82 (95% CI: 1.73–1.92) for tooth loss, and were also associated with higher odds of heart failure (38).

### 4.3 Administrative/claims-based studies

Cho D.H. et al., in their 2020 nationwide retrospective cohort study (Korea), examining 72,971 patients with PD and 72,971 propensity score-matched controls from the NHIS-HEALS database, found that the presence of PD was associated with an approximately 15% increase in the relative risk of developing PAD (HR: 1.15; 95% CI: 1.07–1.23) (33). Therefore, the authors concluded that, in addition to controlling the “classic” risk factors (diabetes, hypertension, dyslipidemia, smoking), prevention and treatment of PD should also have been considered part of PAD prevention strategies (33).

In a 2020 retrospective study based on German statutory health insurance claims (BARMER), Aarabi et al. evaluated whether prior periodontal therapy was associated with PAD severity among hospitalized symptomatic PAD patients (30). Using claims from January 1, 2012 to December 31, 2016, PAD stage was defined via ICD-10 codes as intermittent claudication (IC) or chronic limb-threatening ischemia (CLTI), while periodontal treatment was captured through ambulatory billing codes for non-surgical/surgical periodontal care (including supra- or subgingival debridement) (30). The cohort comprised 70,944 hospitalized PAD patients, of whom 3,567 (5.03%) had received periodontal treatment before hospitalization (30). Treated patients showed a markedly lower proportion of CLTI compared with untreated patients (28.76% vs. 52.12%) (30). In multivariable logistic regression adjusting for age, sex, and diabetes, having CLTI (vs. IC) was associated with not having received periodontal treatment (OR: 1.97, 95% CI: 1.83–2.13) (30). Overall, the analysis suggested an inverse association between prior periodontal treatment and PAD severity, while interpretation remained limited by the use of claims-based proxies for both exposure and outcomes, potential residual confounding, and the inability to establish temporality or exclude reverse causation (30).

A large retrospective observational study conducted by Yeh et al. in 2022 analyzed the incidence of PAD in 357,000 patients with and without PD (37). The study found that PAD occurred somewhat more frequently in individuals with PD, with a higher risk among patients older than 65 years (HR: 3.17; 95% CI: 3.07–3.27) (37). In addition, undergoing at least one session of dental hygiene per year was associated with a lower risk of developing PAD (HR: 0.84; 95% CI: 0.78–0.89), both in subjects with PD and in those without PD (37). Therefore, the authors concluded that maintaining healthy gums and good oral hygiene might also have conferred benefits for the health of the blood vessels in the legs (37).

### 4.4 Murine studies

In an experimental mouse-model study conducted by Zhu et al. in 2025, it was concluded that PD altered the microbiota of the femoral artery (with a relative increase in anaerobes compared with aerobic bacteria) and, at the same time, modified the arterial immune environment, with a central role played by FABP4-positive endothelial cells (45). These cells acted as sensors of microbial signals, recruited innate immune cells, and promoted the differentiation of T cells toward proinflammatory phenotypes (45). FABP4-positive endothelial cells were therefore proposed as key mediators of microbiota–immunity crosstalk in peripheral arteries and as a potential therapeutic target in PAD associated with PD (45). However, the study had limitations related to the use of a murine model and to the differences between the murine and human microbiota (45).

### 4.5 International consensus report

The international consensus report conducted by Sanz M. et al. summarized the evidence on the relationship between PD and CVD and provided recommendations for dentists, physicians, and patients (54). In this context, based on two large population-based cross-sectional studies from the U.S. (NHANES 1999–2002) and South Korea (KoGES-CAVAS) and one long-term cohort of U.S. veterans, PAD was confirmed to be more frequent among individuals with PD than among those without PD, with adjusted OR of 2.2 (95% CI: 1.2–2.4) and 2.0 (95% CI: 1.1–3.9) for ABI-defined PAD in the cross-sectional studies, and an OR of 2.3 (95% CI: 1.3–3.9) for incident PAD in the prospective veteran cohort (54). The link between PD and CVD was supported by the evaluation of several parameters, including oral bacteraemia, the presence of periodontal bacteria in atherosclerotic plaques, systemic inflammation, altered thrombotic and lipid profiles, hyper-reactive neutrophil responses, autoimmunity and molecular mimicry, and shared genetic factors (54). From the analysis of multiple observational cohort studies, it emerged that regular oral hygiene, periodic dental visits, and periodontal therapy were associated with a reduced incidence of atherosclerotic cardiovascular events (54). The authors therefore concluded that dentists should not only have informed patients with PD about its association with CVD, but also emphasize the need for frequent hygiene sessions and periodontal therapy, and educate them on meticulous home oral care (54). In addition, patients with CVD should have undergone comprehensive periodontal examinations and, even in the absence of PD, should have been included in a preventive program with at least annual check-ups (54). In cases of PD in patients with CVD, dental hygiene sessions should have been short and repeated, in order to limit peaks of systemic inflammation (54). Conversely, internists, cardiologists, general practitioners, cardiothoracic surgeons, and vascular surgeons should have informed patients that PD may worsen their cardiovascular health and referred them to their dentist (54). However, the consensus report by Sanz M. et al. does not include the most recent evidence provided by studies published after 2020, including those by Zhu et al., Yu et al., and Yeh (54).

## 5 Conclusions

The literature on the association between PD and PAD reported heterogeneous findings, yet overall supported a modest link between the two conditions (54). These data nonetheless underscored the importance of oral health in the prevention of CVD. Concepts well established regarding the association between periodontal bacteria and cardiovascular tissues might have extended to arterial disease of the peripheral vessels. However, several barriers and confounders still prevented precise quantification of this association, enough to ascribe prognostic value to PD for the onset of PAD. Future large-scale, longitudinal and interventional studies are needed to clarify whether timely diagnosis and treatment of PD can translate into a tangible reduction in PAD incidence and progression, and to better define the clinical relevance of incorporating periodontal status into vascular risk stratification.
